# Essential roles of leucine-rich glioma inactivated 1 in the development of embryonic and postnatal cerebellum

**DOI:** 10.1038/srep07827

**Published:** 2015-01-16

**Authors:** Ya-Jun Xie, Liang Zhou, Nanwei Jiang, Nan Zhang, Na Zou, Lin Zhou, Yin Wang, John K. Cowell, Ying Shen

**Affiliations:** 1 Department of Neurobiology, Key Laboratory of Medical Neurobiology of the Ministry of Health, Zhejiang Province Key Laboratory of Neurobiology, Zhejiang University School of Medicine, Hangzhou, China; 2 Zhejiang Provincial Key Laboratory of Pathophysiology, Department of Physiology and Pharmacology, Ningbo University School of Medicine, Ningbo, China; 3 Ningxia Key Laboratory of Cerebrocranial Diseases, Ningxia Medical University, Yinchuan, China; 4 Georgia Regents University, Cancer Center, Augusta, GA, USA

## Abstract

Leucine-rich glioma inactivated 1 (LGI1) is a secreted protein that interacts with ADAM transmembrane proteins, and its mutations are linked to human epilepsy. The function of LGI1 in CNS development remains undefined. Here, we report novel functions of LGI1 in the generation of cerebellar granule precursors (CGPs) and differentiation of radial glial cells (RGCs) in the cerebellum. A reduction in external granule layer thickness and defects in foliation were seen in embryonic and new-born LGI1 knockout (KO) mice. BrdU staining showed an inhibited proliferation of CGPs in KO embryos, which might be explained by the reduced Sonic hedgehog in embryos. In addition, the differentiation of RGCs into Bergmann glias was suppressed in KO mice. Enhanced Jagged1-Notch1 signaling in KO mice *via* reduced β-secretase proteolysis suggests that altered phenotype of RGCs is due to abnormal Notch1 signaling. Together, our results demonstrate that LGI1 is an essential player in the cerebellar development.

Leucine-rich glioma inactivated 1 (*Lgi1*) is a human epilepsy-related gene^1^ that encodes a secreted neuronal protein in the central nervous system (CNS)^2^,^3^. Mutations in LGI1 are responsible for autosomal dominant lateral temporal lobe epilepsy (ADLTE), an inherited syndrome characterized by seizure and hallucinations^4^. The secretion of LGI1 is prevented in cultured cells from ADLTE patients carrying mutations, suggesting that LGI1 haploinsufficiency is the pathogenic basis for ADLTE^3^,^5^. Consistent with the human seizure-prone disease, LGI1-knockout mice display generalized seizures and die within 3 weeks after birth^6^,^7^,^8^.

Although LGI1 is shown to be correlated with epilepsy, its precise functions in the CNS are still poorly understood. Existing evidence shows that LGI1 prevents the inactivation of the voltage-gated potassium channel Kv1^9^ and regulates the pruning and synaptic transmission of glutamatergic synapses in the hippocampus^5^,^7^,^10^. The refined synaptogenesis might be mediated by the binding of LGI1 to transmembrane ADAM (a disintegrin and metalloproteinase) family proteins (ADAM22, ADAM23, and ADAM11). These proteins stabilize α-amino-3-hydroxy-5-methyl-4-isoxazole-propionic acid receptors at the neuronal surface and thus modulate synaptogenesis^5^,^11^. LGI1 also directly binds to Nogo receptor 1 (NgR1) and ADAM22 simultaneously to form a receptor complex, in which NgR1 facilitates the association between LGI1 and ADAM22^12^. Despite these findings, the function of LGI1 in CNS development remains largely undefined. In fact, previous work has shown that LGI1 is expressed in the cerebellum and Purkinje cells (PCs) in the embryonic and postnatal periods^13^,^14^.

It has been suggested that LGI1 affects other receptors or intracellular signaling cascades because it overrides the inhibition of neuronal growth by Nogo66^12^. In addition, LGI1 contains a putative nuclear localization signal that leads to its nuclear location in the caudal ganglionic eminence neurons of the early embryonic telencephalon^15^. Therefore, it is possible that, in addition to epilepsy, LGI1 is involved in nuclear mitogenic signaling, migration and morphology, and cell differentiation in the CNS. Here, we report unexpected roles of LGI1 in the early development of the mouse cerebellum. In embryonic and new-born LGI1-knockout mice, a reduction in the thickness of the external granule layer (EGL) as well as foliation defects is seen. In addition, the postnatal differentiation of radial glial cells (RGCs) into Bergmann glias (BGs) is suppressed in postnatal knockout mice. Molecular studies suggest that these altered phenotypes are due to abnormal Sonic hedgehog (Shh) and Notch signaling during cerebellar development in LGI1-knockout mice.

## Results

### Expression of LGI1 in the cerebellum and phenotypes of LGI1-knockout mice

We assessed the expression levels of LGI1 protein in the cerebellum during developmental and postnatal periods. Identical amounts of protein from cerebellar homogenates of mice at different developmental stages were resolved by SDS-PAGE and immunoblotted with the LGI1 antibody. LGI1 was expressed abundantly in pups from E12.5 to 2 months (supplementary Fig. S1a). While the expression levels were constant during the embryonic stages, they increased rapidly from P14 (supplementary Fig. S1a).

The *Lgi1*-null mutant mouse (KO) used in the present work was created by deleting the genomic region of *Lgi1* extending from exon 3 to exon 8^8^. This mutation strategy efficiently reduces the probability of generating truncated LGI1 proteins^8^. The mutation was confirmed by PCR and western blotting (supplementary Fig. S1b). In addition, the wild-type (WT) had pink eyes and a white coat, while the KO mice had dark ruby eyes and a grey coat (supplementary Fig. S1c). The different coat colors occur because of the tyrosinase marker included in the KO vector^8^. Although KO mice appeared normal at birth, they were subject to weight loss from P5, as shown by the body size at P7 and P14^6^ (supplementary Fig. S1c). Similar to previous work^16^, P14 KO mice showed clasping behavior in the tail-suspension test (supplementary Fig. S1d), suggesting abnormal motor behavior^17^. The mutant mice had a slightly smaller cerebellum at P14 (supplementary Fig. S1e). KO mice typically demonstrated seizure phenotypes after ~17 days, and died shortly afterwards^8^.

### Delayed formation of folia in KO cerebellum

**Figure 1:**
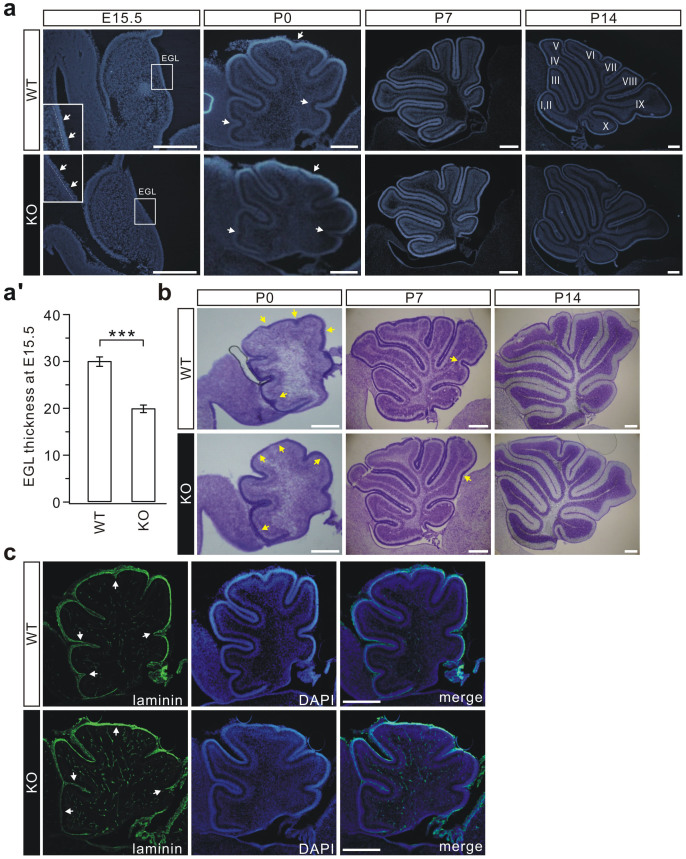
Abnormal foliation in the KO cerebellum. (a) Mid-sagittal sections of E15.5, P0, P7, and P14 cerebella derived from WT and KO littermates were stained with DAPI. Higher magnifications show segments of the EGL (boxes in E15.5 panels). The dashed lines show the interior edge of EGL. Note that the EGL thickness was reduced in KO cerebella (white arrowheads). At P0, the WT cerebellum had developed 7 lobes, but the KO cerebellum displayed only 5 (white arrowheads). No folia defects were found in P7 and P14 KO littermates. Roman numerals denote cerebellar lobules for both WT and KO cerebella. n = 7 pairs for P0, P7, or P14. Scale bars: 200 μm. (a') The thickness of the EGL at E15.5 was 30.1 ± 1.0 μm (WT) or 20.0 ± 0.8 μm (KO). n = 6 pairs, *** p < 0.001 (p = 0.0003, two-sided Student's *t*-test). (b) H&E staining of mid-sagittal sections from P0, P7, and P14 cerebella. KO cerebella (P0) showed marked agenesis of foliation (arrowheads), which was mostly corrected in P7 and P14 cerebella. Missing fissures were sometimes observed in P7 cerebella (arrowhead in the middle panel). n = 5 pairs for each age. Scale bars: 200 μm. (c) P0 cerebella stained with DAPI and with laminin-1 (laminin). While the foliation deficit was evident in the KO mice (arrowheads in left panels), laminin-1 staining was intact and continuous along the cerebellar surface (n = 5 pairs). Scale bars: 200 μm.

The morphogenesis of the KO cerebellum was studied in sagittal sections of the cerebellum at embryonic and postnatal stages. Interestingly, while the cerebellar morphology was grossly normal, we found an apparent reduction of EGL thickness in the E15.5 KO embryos (Fig. 1a-a'). This finding implies that loss of LGI1 affects cerebellar foliation, because cerebellar granule precursors (CGPs) migrate outwardly to form the EGL^18^, and the expansion of the EGL is necessary for proper foliation^19^,^20^. Indeed, a prominent foliation defect was found in all new-born KO mice examined. At P0, the WT cerebellum developed elongated fissures and lobules. In contrast, some fissures in the KO cerebellum at the same age were less developed or missing, resulting in only 5 cardinal lobes rather than 7 (Fig. 1a). Moreover, the length of the lobes was generally shorter in the KO embryos (Fig. 1a). Unexpectedly, the folia were grossly normal in P7 and P14 KO pups (Fig. 1a). H&E staining was also used to examine the foliation in P0, P7, and P14 cerebella, and showed that LGI1-KO only affected the foliation at P0, and not at P7 or P14 (Fig. 1b). Together, these results suggested that the *Lgi1*-null mutation retards, but does not prevent, the folia formation in the cerebellum.

Laminin-1 is involved in the formation of the basement membrane^21^ and is necessary for cell proliferation, migration, and differentiation in embryonic development^22^. To examine the effects of LGI1 on laminin-1, WT and KO cerebella were stained with an anti-laminin-1 antibody at P0. While the fissure and foliation deficits were evident in KO mice, laminin-1 staining was continuous along the surface (Fig. 1c), indicating that its expression is normal and the basement membrane of the cerebellum is intact.

### Nestin expression is not affected by the Lgi1-null mutation

**Figure 2:**
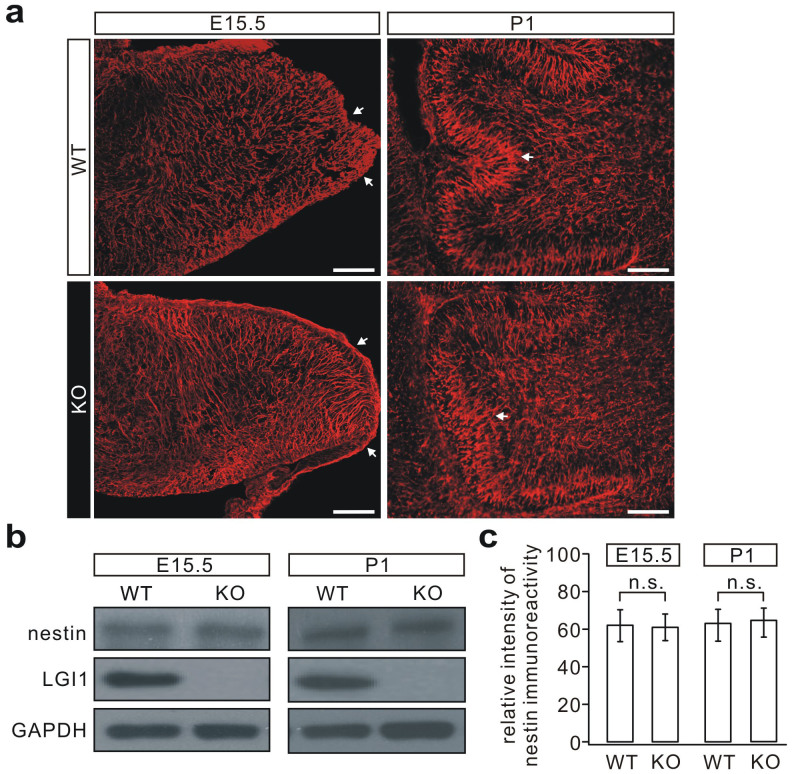
Nestin expression is normal in the KO mice. (a) Nestin staining in the cerebellum (E15.5) and cerebellar lobules (P1). Arrowheads indicate abnormal EGL thickness and folia formation (n = 7 pairs for each age). Scale bars: 50 μm. (b) Total nestin expression levels were measured by western blotting using GAPDH as the internal control. (c) Quantitation of nestin immunoreactivity. The nestin/GAPDH ratios were 61.7 ± 8.0% (WT, E15.5) and 60.6 ± 6.9% (KO, E15.5) (n = 3, p = 0.34); 62.6 ± 8.0% (WT, P1) and 64.1 ± 7.2% (KO, P1) (n = 3, p = 0.17). Statistical analysis was done by two-sided Student's *t*-test. n.s.: no significance.

Since nestin is a well-known marker of neural stem cells (NSCs), and is expressed in undifferentiated CNS cells during development^23^, we investigated its expression in the cerebellum (E15.5 and P1) using immunocytochemical staining and western blotting. Confocal microscopy showed that nestin immunostaining in the KO cerebellum was almost identical to that in the WT cerebellum (Fig. 2a). When pooled cerebellar extracts were used to measure the total expression of the nestin protein (Fig. 2b), consistently, western blotting showed no difference in the nestin expression between WT and KO mice (Fig. 2c).

### Effects of LGI1 on Pax6 expression and granule cell proliferation

**Figure 3:**
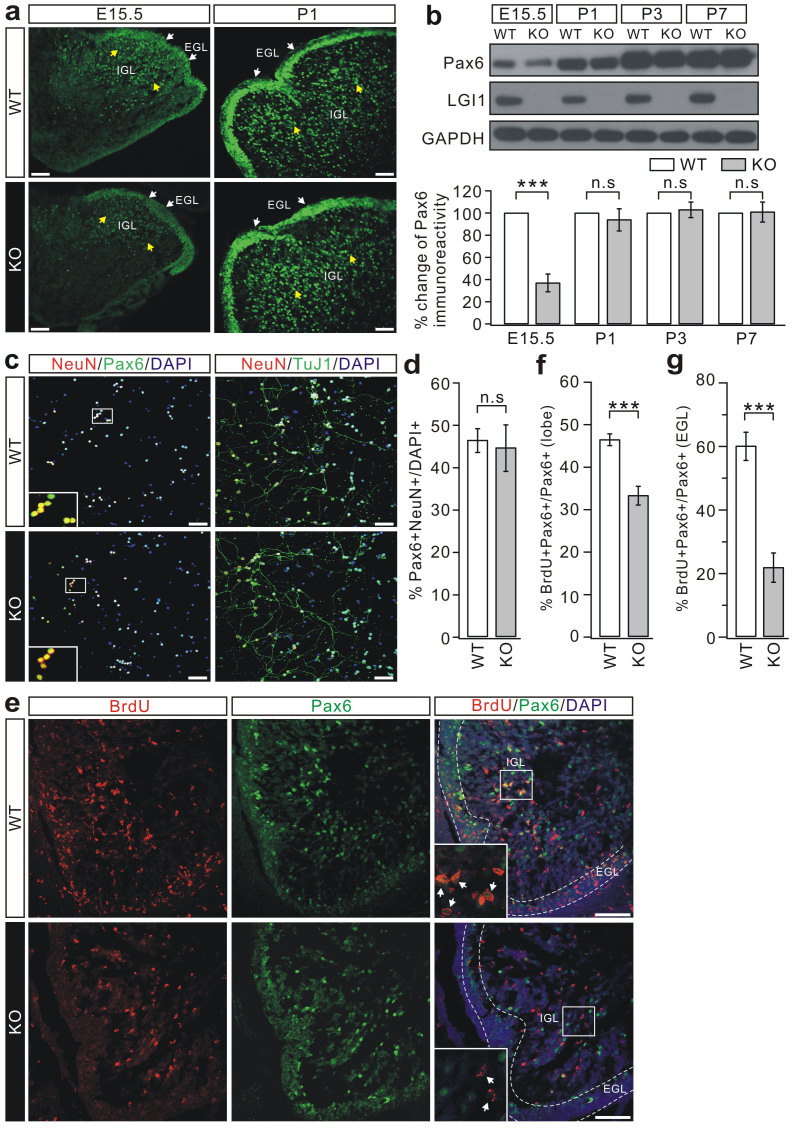
CGP proliferation is reduced in KO mice. (a) Mid-sagittal sections of cerebella stained for Pax6. White and yellow arrowheads indicate Pax6 staining in the EGL and IGL, respectively. Pax6 intensity was reduced at E15.5 (n = 8 pairs), but unchanged at P1 (n = 7 pairs). Scale bars: 50 μm. (b) Total Pax6 expression was measured at E15.5, P1, P3, and P7, using GAPDH as the loading control. The percentage changes of Pax6 (KO/WT) were 36.3 ± 7.9% (E15.5; n = 5 pairs, p = 0.00021), 92.8 ± 9.9% (P1; n = 4 pairs, p = 0.35); 101.7 ± 6.9% (P3; n = 4 pairs, p = 0.32); 99.7 ± 8.9% (P7; n = 4 pairs, p = 0.43). Statistical analysis was done by one-sided Student's *t*-test. *** p < 0.001. n.s.: no significance. (c) Left panels show the staining of NeuN (red), Pax6 (green), and DAPI (blue) in cultured granule cells (DIV12). Higher magnification in white boxes, where the DAPI signal was removed, shows cells double-stained with NeuN and Pax6, indicating that 100% of NeuN-positive cells were Pax6-positive. Right panels: DIV12 granule cells labeled with TuJ1 (green) and NeuN (red). Nuclei were counterstained with DAPI (blue). Immunoreactivity for TuJ1 displayed long processes from differentiated neurons in both groups. Scale bars: 50 μm. (d) Numbers of NeuN+Pax6+ cells in cultured granule cells (DIV12) expressed as percentages of DAPI+stained cells (WT: 46.3 ± 2.8%; KO: 44.5 ± 5.5%; n = 6 pairs) Two-sided Student's *t*-test. p = 0.34. n.s.: no significance. (e) Mid-sagittal sections of P0 cerebella were stained with Pax6, BrdU, and DAPI. Dashed lines define the EGL. Higher magnifications in the white boxes, with the DAPI signal removed, show cells stained by BrdU and Pax6 (arrowheads). Scale bars: 50 μm. (f) Percentages of BrdU+Pax6+ cells/Pax6+ cells in a lobe. n = 5, *** p < 0.001 (p = 0.00012, two-sided Student's *t*-test). (g) Percentages of BrdU+Pax6+ cells/Pax6+ cells within the EGL. n = 5, * p < 0.001 (p = 0.00018, two-sided Student's *t*-test).

To investigate whether the reduction of EGL thickness was due to altered neuronal differentiation, we studied paired-box homeodomain transcription factor (Pax6) expression in the cerebellum. Pax6 expression begins ~E8.5 in the mouse brain^24^ and promotes neuronal differentiation, migration, and neurite extension^25^,^26^. Immunocytochemical staining and western blotting analysis showed that Pax6 was robustly expressed at E15.5 and P1 in WT mice (Fig. 3a), representing CGPs in the EGL and migrating granule cells in the inner granular layer (IGL)^20^,^27^. Pax6 immunoreactivity was much stronger at P1 and displayed a thickened appearance in the EGL (Fig. 3a), similar to previous descriptions^20^,^27^. In the E15.5 KO cerebellum, however, there was a substantial decrease of Pax6 intensity throughout the cerebellum, including the EGL and IGL (Fig. 3a). Interestingly, this decrease in Pax6 immunolabeling was not seen after birth (P1, Fig. 3a), suggesting that the LGI1 deficiency only suppresses Pax6 expression in embryos. Western blotting analysis also confirmed that the total expression of Pax6 was substantially reduced in KO cerebella from E14.5-E16.5 (Fig. 3b, supplementary Fig. S2), but not in cerebella at E17.5 (supplementary Fig. S2) and postnatal P1 to P7 (Fig. 3b). These results implied that LGI1 influences the generation of CGPs during early embryogenesis.

To further study the effects of LGI1 on neuronal development, simultaneous staining with DAPI, Pax6, and NeuN was performed in dissociated cultures of postmitotic granule cells. The proportion of Pax6+NeuN+ cells to DAPI+ cells was calculated as an estimate of the number of NSCs that had differentiated into neurons. There was no statistical difference between WT and KO cultures at DIV12 regarding the number of differentiated granule cells (Fig. 3c–d). Using TuJ1 staining, we also found no difference in either the pattern of tubulin distribution or cellular morphology between WT and KO cultures (Fig. 3c). These data argued that LGI1 is not required for late-phase neuronal maturation.

To determine whether reduced Pax6 was related to CGP proliferation, 5-bromo-2-deoxyuridine (BrdU) staining was analyzed with the incorporation of Pax6 and DAPI in granule cells. To ensure the labeling of dividing cells, a single dose of BrdU was applied daily to pregnant mice from E18 to birth and sagittal sections of the cerebellum were acquired at P0 for confocal imaging. KO cerebella displayed a considerable decrease in the BrdU signal, which was more prominent in the EGL, where limited BrdU signal was seen (Fig. 3e). To quantify the change, labeling indices (BrdU+Pax6+/Pax6+ cells) were assessed in the entire lobule and within the EGL (Fig. 3f-g). In WT mice, the indices were 46 ± 1% in the lobule and 60 ± 4% within the EGL (n = 7), and in KO mice, the indices were 33 ± 2% in the lobule and 22 ± 5% within the EGL (n = 7). Therefore, it appeared that the proliferation of embryonic CGP is suppressed following the loss of LGI1 expression.

### Shh signaling is down-regulated in KO mice

**Figure 4:**
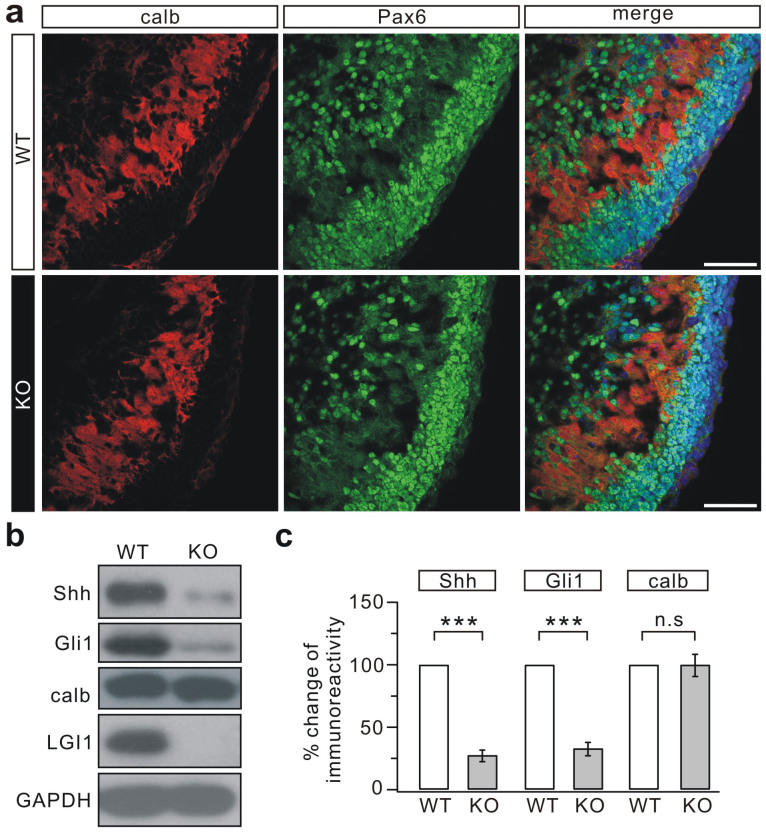
LGI1-deficiency correlates with reduced expression of Shh *in vivo*. (a) Mid-sagittal sections of P1 cerebella stained for calbindin (calb) and Pax6. Note that calbindin labeling was seen in the inner layer whereas the majority of the Pax6 signal was in the outermost layer. Calbindin staining was grossly normal in the KO cerebellum (n = 7 pairs). Scale bars: 50 μm. (b) Total expression of Shh and Gli1 was decreased in the E15.5 KO cerebellum, but calbindin (calb) was not affected. GAPDH was the internal control. (c) The percentage changes of signal intensity (KO/WT) were 26.3 ± 7.9% (Shh; p = 0.0006), 31.7 ± 9.3% (Gli1; p = 0.0008), and 98.1 ± 16.3% (calb; p = 0.24). Experiments were performed on 5 pairs of littermates. Statistical analysis was done by one-sided Student's *t*-test. *** p < 0.001. n.s.: no significance.

Shh protein, made by PCs^28^, is required for CGP generation and proper lobe growth^29^,^30^. We therefore examined the morphology of PCs and Shh expression in KO mice. Double-staining with calbindin and Pax6 showed that the PC layer, which is beneath the EGL and parallel to the laminae, was well-developed in the KO cerebellum at P1 (Fig. 4a). The total expression of calbindin and Shh was measured in the embryonic cerebellum from E14.5-E17.5, and this showed that the expression of Shh and Gli1, a downstream effector of Shh, were markedly reduced in the KO mice, although calbindin expression was not changed (Fig. 4b–c, supplementary Fig. S3). The reduced Shh might explain, at least in part, the inhibited generation of CGPs.

### LGI1-knockout inhibits the differentiation of RGCs

**Figure 5:**
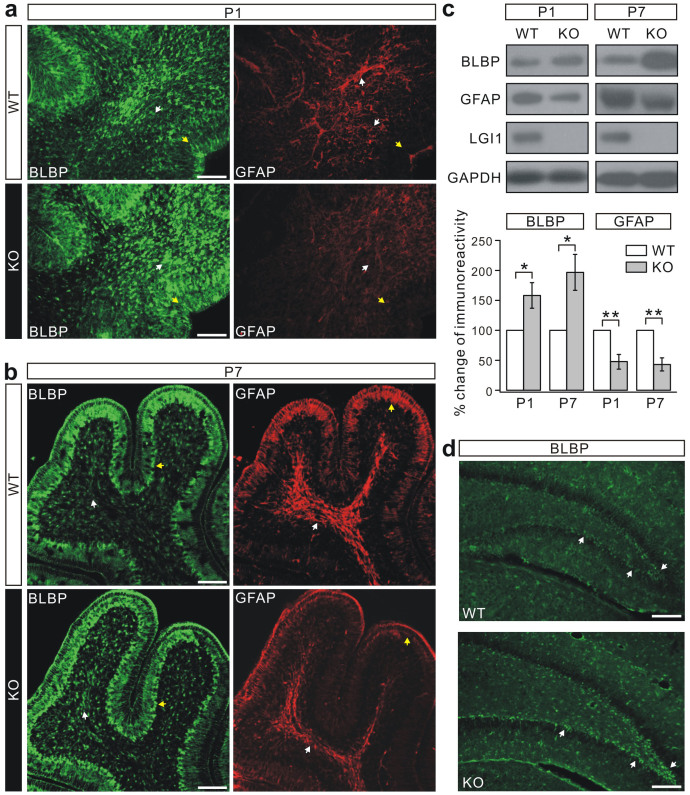
Loss of LGI1 results in reduced differentiation of RGCs. (a) Mid-sagittal sections of P1 cerebella stained for BLBP and GFAP. White and yellow arrowheads indicate the fluorescence in the interior area and EGL respectively, showing that BLBP expression was increased whereas GFAP expression was reduced in the KO mice (n = 5 pairs). Scale bars: 50 μm. (b) Mid-sagittal sections of P7 cerebella stained for BLBP and GFAP. White and yellow arrowheads indicate the fluorescence in the white matter area and BG cell bodies, respectively (n = 5 pairs). Scale bars: 50 μm. (c) Total expression of BLBP and GFAP was measured in WT and KO littermates at P1 and P7 using GAPDH as the internal control. Lower panel: the percentage changes of BLBP and GFAP signal intensity (KO *vs* WT) were 157.5 ± 21.2% (P1, BLBP; p = 0.044), 195.8 ± 29.8% (P7, BLBP; p = 0.028), 47.6 ± 12.2% (P1, GFAP; p = 0.0087), and 42.7 ± 10.8% (P7, GFAP; p = 0.0075). Experiments were performed on 6 pairs of littermates. Statistical analysis was done by one-sided Student's *t*-test. ** p < 0.01. * p < 0.05. (d) Dentate gyrus of P14 hippocampus stained for BLBP. Arrowheads indicate the fluorescence intensity of BLBP in WT and KO mice (n = 3 pairs). Scale bars: 50 μm.

In the cerebellum, RGCs provide a scaffold for the migration of granule cells and are essential for organizing the laminated structure^31^. The disrupted morphogenesis in KO mice led us to determine whether the phenotype of RGCs was affected. Immunostaining for brain lipid-binding protein (BLBP), a specific marker that labels RGCs in the postnatal cerebellum^32^,^33^, showed that RGC fibers were oriented parallel to each other and perpendicular to the outer surface (Fig. 5a), particularly near the anchoring center where CGPs accumulate (Fig. 5a, yellow arrowheads). Unexpectedly, a remarkable increase, rather than a decrease, in the BLBP signal was observed in both the EGL and the IGL of the KO cerebellum (Fig. 5a). At P7, the augmented BLBP staining remained extensive mainly in the cell bodies and white matter area (Fig. 5b). The orientation of RGC fibers was also normal in the KO cerebellum (Fig. 5b).

BLBP-labeled RGCs are transiently present in the developing cerebellum and differentiate into BG by the end of neuronal migration^32^,^33^,^34^. To determine whether the increased expression of BLBP is due to altered gliogenesis, we analyzed the staining pattern of the astrocyte-specific marker glia fibrillary acid protein (GFAP), which showed a considerable reduction of signal in the KO cerebellum, more profoundly in the white matter (Fig. 5a–b). Western blots showed that BLBP expression was unaltered in embryonic cerebella from E14.5-E16.5 (supplementary Fig. S4), but was increased at E17.5 (supplementary Fig. S4), P1 and P7 (Fig. 5c). Meanwhile, GFAP expression was decreased at both P1 and P7 (Fig. 5c). These results suggested that loss of LGI1 function might interrupt the differentiation of RGC to BG.

BLBP is important for the differentiation of immature RGCs into astrocytes in the hippocampal dentate gyrus^35^. Thus, BLBP staining was performed in the hippocampus of P14 mice. Similar to the cerebellum, the intensity of BLBP staining was substantially increased in the molecular layer of the dentate gyrus of KO mice (Fig. 5d).

### Loss of LGI1 decreases the number of BGs *in vitro*

**Figure 6:**
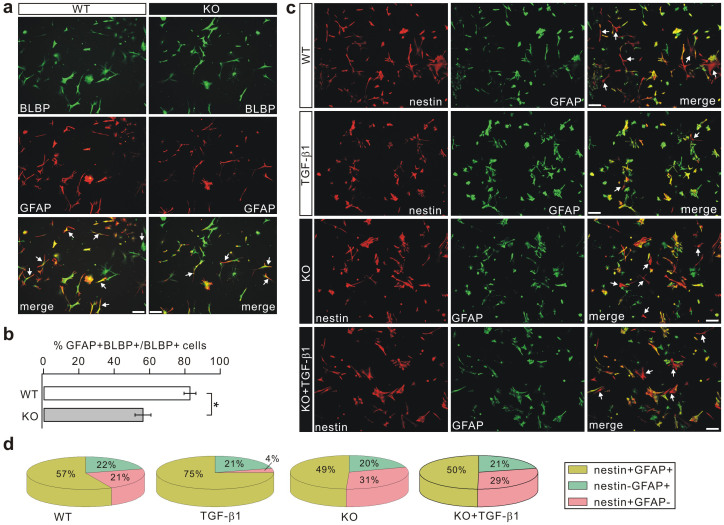
Gliogenesis is decreased in cultured KO RGCs. (a) Cultured RGCs (DIV12) identified by their bipolar morphology and BLBP immunoreactivity. Arrowheads indicate cells double-stained with BLBP and GFAP. Scale bars: 50 μm. (b) KO cultures displayed a decrease in the ratio of GFAP+BLBP+ cells to BLBP+ cells. The percentages were 82.9 ± 3.4% (WT) and 56.2 ± 4.5% (KO), n = 7, * p < 0.05 (p = 0.027, two-sided Student's *t*-test). (c) RGC cultures (DIV12) maintained in medium supplemented with either serum or 10 ng/ml TGF-β1 and stained with nestin and GFAP. Arrowheads indicate nestin+GFAP+ cells. Scale bars: 50 μm. (d) Percentages of each cell type from experiments as in (c).

To further characterize gliogenesis, we turned to acutely-isolated RGC cultures to quantitate the number of GFAP-immunoreactive cells. DIV12 RGC cultures were double-stained with GFAP and BLBP and gliogenesis was evaluated by calculating the relative proportion of GFAP+ cells among the BLBP+ cells. A larger proportion of GFAP+BLBP+ cells was found in the WT group, compared with a substantial reduction in their number in the KO cultures (Fig. 6a–b).

The differentiation of RGCs *in vitro* was also investigated following transforming growth factor-β1 (TGF-β1) activation^36^,^37^. TGF-β1 (10 ng/ml) was added to the medium and DIV12 cells were double-stained with GFAP and nestin to evaluate the cellular composition (Fig. 6c). In WT cultures, the cell types were RGCs (nestin+GFAP-, 21%), BGs (nestin+GFAP+, 57%), and mature astrocytes (nestin-GFAP+, 22%) (Fig. 6d). Mature astrocytes are derived from spontaneously differentiated NSCs^38^,^39^, in which nestin levels are gradually reduced during differentiation and maturation^40^,^41^. TGF-β1 treatment yielded substantial changes in the relative cell composition by reducing the number of RGC (4%) and increasing the number of BG (75%) (Fig. 6d). No change was found in mature astrocytes (21%).

Next, KO RGCs were cultured in normal medium or in medium supplemented with TGF-β1, followed by double-staining for GFAP and nestin; this showed that the percentages of the cell populations were 31% RGC, 49% BG, and 20% mature astrocyte (Fig. 6d). Similar to GFAP-labeling (Fig. 6a), the percentage of BG (nestin+/GFAP+) was lower in the KO cultures than that in WT cells (Fig. 6d). TGF-β1 treatment failed to substantially change the cell populations: 29% RGC, 50% BG, and 21% mature astrocyte (Fig. 6c–d).

### LGI1-deficiency boosts canonical Notch signaling

**Figure 7:**
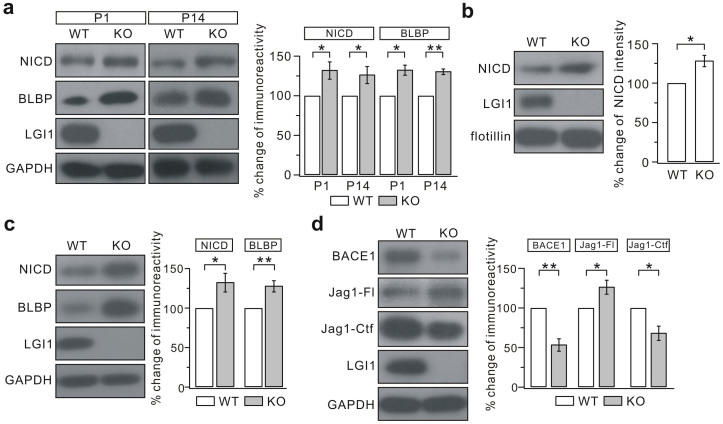
Jagged1-Notch1 signaling is enhanced in KO mice. (a) Expressions of NICD and BLBP in the KO cerebella were increased at P1 and P14. The percentage changes (KO/WT) were 131.8 ± 10.9% (NICD, P1; p = 0.034), 125.3 ± 10.7% (NICD, P14; p = 0.033), 131.4 ± 6.3% (BLBP, P1; p = 0.013), and 129.4 ± 3.5% (BLBP, P14; p = 0.0055). Experiments were performed on 7 pairs of littermates. Statistical analysis was done by one-sided Student's *t*-test. ** p < 0.01. * p < 0.05. (b) NICD expression in the membrane fraction was increased in the P14 KO cerebellum. Flotillin-1 was used as the internal control. The percentage change (KO/WT) was 126.5 ± 7.1%, n = 7, * p < 0.05 (p = 0.013, one-sided Student's *t*-test). (c) Protein levels of NICD and BLBP were increased in KO RGC cultures. The percentage changes (KO/WT) were 131.0 ± 11.7% (NICD; p = 0.026) and 126.4 ± 7.0% (BLBP; p = 0.0094). Experiments were performed on 7 pairs of littermates. Statistical analysis was done by one-sided Student's *t*-test. ** p < 0.01. * p < 0.05. (d) The amount of full-length Jagged1 (Jag1-Fl) were increased, while BACE1 and Jag1-Ctf were decreased, at P3 in KO mice. The percentage changes (KO/WT) were 52.5 ± 7.8% (BACE1; p = 0.0076), 125.3 ± 8.8% (Jag1-Fl; p = 0.023), and 67.2 ± 8.9% (Jag1-Ctf; p = 0.015). Experiments were performed on 4 pairs of littermates. Statistical analysis was done by one-sided Student's *t*-test. ** p < 0.01. * p < 0.05.

BLBP protein is a direct target of Notch signaling^42^ and active Notch1 signaling is critical for maintaining the proper phenotype of RGCs^43^,^44^,^45^. To investigate if suboptimal Notch1 signaling is responsible for the altered phenotype of RGCs in the KO cerebellum, the canonical Notch1 signaling pathway was examined. Western blotting showed a considerable increase in the expression of Notch1 intracellular domain (NICD), which is generated by Notch1 activation^46^, in KO cerebella at both P1 and P14 (Fig. 7a). However, the expression of NICD was not changed in embryonic stages from E14.5-E17.5 (supplementary Fig. S4), suggesting that Notch1 signaling is up-regulated in LGI1-deficient mice in a timing-dependent manner. This conclusion was supported by two lines of additional evidence. First, NICD enriched in the purified membrane fraction was increased in the P14 KO cerebellum (Fig. 7b). Second, the expression of NICD was elevated in acutely-dissociated DIV12 RGC cultures (Fig. 7c).

Notch activation is mediated by cell-cell interactions between Notch on one cell and its transmembrane-anchored ligands on neighboring cells^47^. The interactions between ligands and Notch receptors trigger the proteolytic cleavage of Notch and the release of NICD^47^. To study how Notch1 becomes activated, we assessed the expression of Jagged1, an essential Notch1 ligand, and found that Jag1-Fl was substantially elevated in the P3 KO cerebellum, while Jag1-Ctf was reduced (Fig. 7d). The increase of Jag1-Fl, which activates Notch1, may explain the increased expression of NICD. Meanwhile, BACE1 (β-site amyloid precursor protein-cleaving enzyme 1, β-secretase), which cleaves Jagged1^47^,^48^, was reduced (Fig. 7d), suggesting that the elevated Jag1-Fl is due to decreased BACE1 activity. Taken together, this enhanced Jagged1-Notch signaling in KO mice appears to be achieved by reduction of the proteolytic processing of Jagged1 by BACE1.

## Discussion

In summary, we report that deletion of LGI1 leads to reduced EGL thickness, disrupted cerebellar foliation, and reduced differentiation of RGC to BG. The defects in EGL thickness and folia formation may be caused by the reduced proliferation of CGPs and the down-regulation of Shh signaling. Inhibition of gliogenesis as demonstrated by increased BLBP and decreased GFAP may be due to enhanced Jagged1-Notch1 signaling *via* reduced proteolytic processing by BACE1. Together, these data demonstrate that LGI1 plays a critical role in the development of the cerebellum.

An interesting finding in this study was that the effects of LGI1 on neuronal development were mainly seen during embryonic development, and not in the young adult. The evidence includes: 1) the foliation defects were seen in embryonic and new-born KO mice, but not in pups after P7; and 2) Pax6 expression was decreased in the E15.5 KO cerebellum, but this decrease was not present after birth. One plausible explanation for these phenomena is that other molecules compensate for the loss of LGI1 and act on its downstream effectors. Since LGI1 is part of a four-member family of conserved proteins^49^ (LGI1, 2, 3, and 4) and some of these may share a common interaction site with ADAM proteins^50^, they may provide compensatory stimulation of the proliferation of CGPs and restore the defects of Pax6 expression and foliation. It is important, therefore, to investigate the expression patterns of the various LGI family members and determine their effects during cerebellar development.

We speculate that the inhibition of Pax6 expression and CGP proliferation in KO embryos is mediated by Shh signaling. The mechanism behind the association between LGI1 and Shh, however, has not been revealed in the present work. One perspective is that the effects of LGI1 may be mediated by ADAM proteins, because LGI1, a secreted protein, mainly exerts its functions through binding ADAMs. It has been shown that ADAM proteins regulate phenotypes such as cell adhesion, migration, and proteolysis^51^. Moreover, their downstream signaling is responsible for the expression of growth factors and cytokines. For example, ADAM17 is a sheddase for the production of tumor necrosis factor α, TGF-α, and p75^52^, suggesting the importance of LGI-ADAM signaling for secretary proteins. Although the production of Shh, a key morphogen as well as a secreted protein, may be potentially affected by a certain ADAM signaling, the association between LGI1 and Shh could be more complicated. Promoter activation in model systems showed that LGI1 is expressed in PCs^13^. Another work by Kunapuli et al.^53^ showed that the forced re-expression of Lgi1 in glioma cells, which are thought to be derived from neuronal precursor cells, results in an up-regulation of Gli2. These results suggest an idea that Lgi1 protein may also regulate the Shh signaling through intracellular mechanisms.

The activation of Notch signaling is mediated by the interaction between Notch receptors and their ligands. In mammals, the main ligands for Notch are Jagged1, Jagged2, DLL1, DLL3, and DLL4^54^. Among these ligands, only Jagged1 has the ability to activate Notch *via* cell-cell juxtacrine signaling and the ectodomain shedding of Jagged1 downregulates its activity^47^. Recently, Hu et al.^48^ determined the relationship between BACE1 and Jagged1 by showing that overexpression of BACE1 enhances the cleavage of Jagged1, and shedding of Jagged1 is reduced in BACE1-null mice. They also demonstrated that BACE1-null mice exhibit a significant increase in RGCs with a decrease in neurogenesis during early development of the hippocampus^48^. In agreement with their results, we found that LGI1-deficiency enhanced the Jagged1 level and reduced BACE1 (Fig. 7). Meanwhile, neurogenesis in the EGL was decreased (Fig. 1) and BLBP-labeled RGCs increased in LGI1-knockout mice (Fig. 5). All these findings indicate that BACE1 functions as a signaling protease that controls the balance of neurogenesis and gliogenesis *via* the juxtacrine Jagged1-Notch pathway. Moreover, our data showed that LGI1 may be a critical upstream regulator of this juxtacrine signaling. Prevailing evidence has shown that Notch activity is regulated by nuclear effectors, receptor proteolysis, glycosyltransferase modifiers, membrane trafficking regulators, and NICD degradation^47^,^54^. Ligand binding leads to the cleavage of Notch by ADAM10 (α-secretase) and ADAM17 (γ-secretase) and the release of NICD^47^,^54^. Our work proposes the new perspective that LGI1 modulates Notch signaling *via* β-secretase (BACE1), except α-secretase and γ-secretase,

Over the last decade, LGI proteins have emerged as essential regulators of cell-cell interactions in the central and peripheral nervous system. Mutations in LGI proteins have been associated with diverse pathologies such as epilepsy, psychiatric disorders, and hypomyelination^4^. However, their mechanisms of action are poorly understood. Studying this interesting family is critical for understanding the proper development of the vertebrate nervous system and for gaining insights into therapies for diseases associated with them. In this sense, our work provides a new insight into the functions of LGI proteins in the development of the cerebral cortex, although it was performed in the cerebellum. It is known that all LGI proteins interact with ADAM22/23/11 receptors and may act through a similar mechanism^4^,^50^. However, it is not known whether LGI proteins are functionally equivalent or serve distinct functions in different parts of the CNS at different developmental stages. One published work showed that LGI3 cannot replace LGI1 in synaptic development^7^. Interestingly, we showed that the differentiation of RGCs remained impaired but neurogenesis and foliation were restored after P7 in LGI1-KO mice. These data suggest that LGI proteins may be not functionally equivalent: on one hand, one or more LGI proteins may also promote neurogenesis; on the other hand, LGI1 can modulate Notch1 signaling and RGC differentiation but other family members cannot. Looking ahead, investigations to identify the functional relationships between the LGI protein repertoire and ADAM receptors as well as the downstream cascades of LGI-ADAM interactions in different parts of the CNS, may ultimately elucidate the mechanistic aspects that are either common to the LGI protein family or specific to individual members.

## Methods

### Animals

All experiments protocols were approved by the Animal Experimentation Ethics Committee of Zhejiang University and were specifically designed to minimize the number of animals used. Experiments with animals were carried out in accordance with the guidelines of the Animal Care and Use Committee of the Zhejiang University School of Medicine and the NIH Guide for the care and use of laboratory animals. Original breeding pairs of the LGI1-knockout strain were obtained from John Cowell (Georgia Regents University) and maintained at the Experimental Animal Center of Zhejiang University. Mice were kept under temperature-controlled condition on a 12:12 h light/dark cycle with food and water *ad libitum*.

### Antibodies and reagents

Antibodies against LGI1 and Shh were purchased from Abcam (Cambridge, UK). Antibodies to GFAP, BLBP, nestin, Pax6, NeuN, and GAPDH were from Millipore (Billerica, MA). Antibodies to BACE1, NICD, Gli1, and Jagged1 were from Santa Cruz (Dallas, TX). Antibodies against calbindin and BrdU were from Sigma (St. Louis, MO). Flotillin-1 antibody was from BD Pharmingen (San Jose, CA). Horseradish peroxidase-conjugated secondary antibodies for immunoblotting were from GE Healthcare (Waukesha, WI). Dulbecco's modified Eagle's medium (DMEM), DAPI, Alexa Fluor-conjugated secondary antibodies, Neurobasal medium, and B27 supplements were from Invitrogen (Carlsbad, CA). The protease inhibitor cocktail was from Merck Chemicals (Darmstadt, Germany).

### Granule cell culture

Cultures of dissociated granule cells were prepared as described previously^55^. Cerebellar cortex from anesthetized P1 mice was dissected on ice, digested with 0.1% trypsin for 10 min at 37°C, and dissociated into individual cells by gentle trituration. Cells were plated on coverslips coated with 100 g/ml poly-D-lysine in Neurobasal medium supplemented with 10% fetal bovine serum and 2% B27. Cells were grown in basal modified Eagle's medium containing 10% heat-inactivated fetal calf serum, gentamycin (100 μg/ml), and 5 mM KCl. Culture dishes were incubated at 37°C under humidified 95% O_2_/5% CO_2_. After 18–20 h, 10 μM cytosine arabinoside was added to the medium to prevent the replication of non-neuronal cells. The culture medium was renewed every three days.

### RGC culture

RGCs were purified from P2-4 cerebella as described previously^56^. Dissociated cells were treated with trypsin/EDTA and the cell suspension was applied onto a one-step Percoll gradient (35%). After centrifugation, cells collected from the interface layer were plated on laminin-coated culture dishes. For TGF-β1 treatment, cultures were supplemented with 10 ng/ml TGF-β1 (PeproTech, Rocky Hill, NJ) after the first medium change.

### *In vivo* BrdU pulse

BrdU (4 mg/200 g body weight) was injected intraperitoneally into pregnant female mice as a single daily dose from E18 to birth. Before BrdU staining, cerebellum sections were incubated in 2 N HCl for 30 min and 0.1 M boric acid for 10 min at 37°C.

### H&E staining

Tissue sections were stained with alum hematoxylin for 5–10 min and eosin for 0.5–2 min. The sections were then dehydrated twice with 95% ethanol and twice with 100% ethanol. Each round of dehydration lasted 30 s. After replacing the ethanol with xylene, mounting medium was dropped onto the sections, which were then coverslipped.

### Preparation of plasma membrane fractions

The plasma membrane fraction was prepared as described previously^57^. Cerebellar tissues were gently homogenized in 500 μl buffer (in mM: 250 sucrose, 20 HEPES, 10 KCl, 1.5 MgCl_2_, 1 EDTA, 1 EGTA) supplemented with protease inhibitor. The homogenate was centrifuge at 720 *g* for 5 min at 4°C, and the supernatant was centrifuged again at 8,000 *g* for 10 min at 4°C. The supernatant was further centrifuged at 100,000 *g* for 1 h at 4°C.

### Western blotting

Western blotting was performed as reported previously^58^,^59^,^60^. Proteins derived from cerebellar tissues or cultured cells were rinsed with phosphate-buffered saline (PBS) and diluted in 1% SDS containing protease inhibitor cocktail. Protein concentration was determined using the BCA protein assay (Bio-Rad, Hercules, CA). Equal quantities of proteins were loaded and fractionated on sodium dodecyl sulfate-polyacrylamide gels (SDS-PAGE) and transferred to PVDF membrane (Immobilon-P, Millipore), immunoblotted with antibodies, and visualized by enhanced chemiluminescence (Pierce Biotechnology, Rockford, IL). The primary antibody dilutions used were LGI1 (1:1,000), Shh (1:1,000), Gli (1:1,000), BACE1 (1:1,000), NICD (1:500), Jagged1 (1:1,000), GFAP (1:10,000), BLBP (1:5,000), nestin (1:2,000), calbindin (1:10,000), Pax6 (1:1,000), flotillin-1 (1:10,000), and GAPDH (1:10,000). Film signals were digitally scanned and quantitated using ImageJ 1.42q (NIH, Bethesda, MD).

### Immunohistochemistry and immunocytochemistry

30-μm sagittal sections were prepared and placed in blocking solution (1% BSA, 0.3% Triton, 10% normal goat serum) for 1 h at room temperature (RT). After washing with PBS, sections were incubated with primary antibodies overnight at 4°C and then incubated with secondary antibodies for 1 h at RT. The secondary antibodies Alexa Fluor 488-conjugated goat anti-rabbit IgG and/or Alexa Fluor 594-conjugated goat anti-mouse IgG were diluted at 1:1000. The sections were mounted using ProLong Gold Antifade Reagent with DAPI (Invitrogen).

Cultured cells were fixed with 4% paraformaldehyde for 15 min at RT, washed with PBS and permeabilized in 0.2% Triton X-100 for 10 min, blocked in 10% BSA for 1 h, and labeled with primary antibodies overnight at 4°C, then cells were incubated with a 1:1000 dilution of Alexa Fluor 488-conjugated goat anti-rabbit IgG and/or a 1:1000 dilution of Alexa Fluor 594-conjugated goat anti-mouse IgG for 1 h at RT.

The primary antibody dilutions used for immunohistochemistry and immunocytochemistry were NeuN (1:200), Pax6 (1:1,000), GFAP (1:1,000), BLBP (1:1,000), nestin (1:500), BrdU (1:200), calbindin (1:1,000), and laminin-1 (1:100). All antibodies were diluted in PBS containing 1% BSA and 1% normal goat serum.

### Statistics

Data in the text and figures are presented as mean ± SEM. Data analysis and statistics were performed using Excel 2003 (Microsoft, Seattle, WA), Igor Pro 6.0 (Wavemetrics, Lake Oswego, OR), and SPSS 16.0 statistical program (SPSS, Chicago, IL, USA). Statistical differences were determined using unpaired one-sided or two-sided Student's *t*-test throughout the work. The accepted level of significance was p < 0.05 (α value). “n” represents the number of animals or cultures tested.

## Author Contributions

Y.-J.X. and Y.S. designed research; Y.-J.X., L.Z., N.J., N.Z., N.Z., and L.Z. performed research; Y.W. and J.K.C. contributed unpublished reagents/analytic tools; Y.-J.X., L.Z., and Y.S. analyzed data; Y.-J.X., J.K.C., and Y.S. wrote the paper.

## Supplementary Material

Supplementary Information supplemental figures and legend

## References

[b1] Kalachikov S. *et al.* Mutations in LGI1 cause autosomal-dominant partial epilepsy with auditory features. Nat. Genet. 30, 335–341 (2002). 11810107 10.1038/ng832 PMC2606053

[b2] Gu W., Brodtkorb E. & Steinlein O. K. LGI1 is mutated in familial temporal lobe epilepsy characterized by aphasic seizures. Ann. Neurol. 52, 364–367 (2002). 12205652 10.1002/ana.10280

[b3] Senechal K. R., Thaller C. & Noebels J. L. ADPEAF mutations reduce levels of secreted LGI1, a putative tumor suppressor protein linked to epilepsy. Hum. Mol. Genet. 14, 1613–1620 (2005). 15857855 10.1093/hmg/ddi169

[b4] Kegel L., Aunin E., Meijer D. & Bermingham J. R. LGI proteins in the nervous system. ASN Neuro. 5, 167–181 (2013). 23713523 10.1042/AN20120095 PMC3691968

[b5] Fukata Y. *et al.* Epilepsy-related ligand/receptor complex LGI1 and ADAM22 regulate synaptic transmission. Science 313, 1792–1795 (2006). 16990550 10.1126/science.1129947

[b6] Chabrol E. *et al.* Electroclinical characterization of epileptic seizures in leucine-rich, glioma-inactivated 1-deficient mice. Brain 133, 2749–2762 (2010). 20659958 10.1093/brain/awq171 PMC2929330

[b7] Fukata Y. *et al.* Disruption of LGI1-linked synaptic complex causes abnormal synaptic transmission and epilepsy. Proc. Natl. Acad. Sci. USA 107, 3799–3804 (2010). 20133599 10.1073/pnas.0914537107 PMC2840530

[b8] Yu Y. E. *et al.* Lgi1 null mutant mice exhibit myoclonic seizures and CA1 neuronal hyperexcitability. Hum. Mol. Genet. 19, 1702–1711 (2010). 20130004 10.1093/hmg/ddq047 PMC2850618

[b9] Schulte U. *et al.* The epilepsy-linked Lgi1 protein assembles into presynaptic Kv1 channels and inhibits inactivation by Kvbeta1. Neuron 49, 697–706 (2006). 16504945 10.1016/j.neuron.2006.01.033

[b10] Zhou Y. D. *et al.* Arrested maturation of excitatory synapses in autosomal dominant lateral temporal lobe epilepsy. Nat. Med. 15, 1208–1214 (2009). 19701204 10.1038/nm.2019 PMC2759408

[b11] Sagane K., Ishihama Y. & Sugimoto H. LGI1 and LGI4 bind to ADAM22, ADAM23 and ADAM11. Inter. J. Biol. Sci. 4, 387–396 (2008). 10.7150/ijbs.4.387 PMC2575350 18974846

[b12] Thomas R. *et al.* LGI1 is a Nogo receptor 1 ligand that antagonizes myelin-based growth inhibition. J. Neurosci. 30, 6607–6612 (2010). 20463223 10.1523/JNEUROSCI.5147-09.2010 PMC6632578

[b13] Head K. *et al.* Defining the expression pattern of the LGI1 gene in BAC transgenic mice. Mamm. Genome 18, 328–337 (2007). 17565425 10.1007/s00335-007-9024-6

[b14] Silva J., Wang G. & Cowell J. K. The temporal and spatial expression pattern of the LGI1 epilepsy predisposition gene during mouse embryonic cranial development. BMC Neurosci. 12, 43 (2011). 21569517 10.1186/1471-2202-12-43 PMC3120723

[b15] Kusuzawa S. *et al.* Leucine-rich glioma inactivated 1 (Lgi1), an epilepsy-related secreted protein, has a nuclear localization signal and localizes to both the cytoplasm and the nucleus of the caudal ganglionic eminence neurons. Eur. J. Neurosci. 36, 2284–2292 (2012). 22612501 10.1111/j.1460-9568.2012.08129.x

[b16] Silva J., Sharma S., Hughes B., Yu Y. E. & Cowell J. K. Homozygous inactivation of the Lgi1 gene results in hypomyelination in the peripheral and central nervous system. J. Neurosci. Res. 88, 3328–3336 (2010). 20857514 10.1002/jnr.22496 PMC3885985

[b17] Mangiarini L. *et al.* Exon 1 of the HD gene with an expanded CAG repeat is sufficient to cause a progressive neurological phenotype in transgenic mice. Cell 87, 493–506 (1996). 8898202 10.1016/s0092-8674(00)81369-0

[b18] Miale I. L. & Sidman R. L. An autoradiographic analysis of histogenesis in the mouse cerebellum. Exp. Neurol. 4, 277–296 (1961). 14473282 10.1016/0014-4886(61)90055-3

[b19] Wang V. Y. & Zoghbi H. Y. Genetic regulation of cerebellar development. Nat. Rev. Neurosci. 2, 484–491 (2001). 11433373 10.1038/35081558

[b20] Sudarov A. & Joyner A. L. Cerebellum morphogenesis: the foliation pattern is orchestrated by multi-cellular anchoring centers. Neural. Dev. 2, 26 (2007). 18053187 10.1186/1749-8104-2-26 PMC2246128

[b21] Miner J. H., Li C., Mudd J. L., Go G. & Sutherland A. E. Compositional and structural requirements for laminin and basement membranes during mouse embryo implantation and gastrulation. Development 131, 2247–2256 (2004). 15102706 10.1242/dev.01112

[b22] Yurchenco P. D. & Patton B. L. Developmental and pathogenic mechanisms of basement membrane assembly. Curr. Pharm. Des. 15, 1277–1294 (2009). 19355968 10.2174/138161209787846766 PMC2978668

[b23] Lendahl U., Zimmerman L. B. & McKay R. D. CNS stem cells express a new class of intermediate filament protein. Cell 60, 585–595 (1990). 1689217 10.1016/0092-8674(90)90662-x

[b24] Inoue T., Nakamura S. & Osumi N. Fate mapping of the mouse prosencephalic neural plate. Dev. Biol. 219, 373–383 (2000). 10694429 10.1006/dbio.2000.9616

[b25] Engelkamp D., Rashbass P., Seawright A. & van Heyningen V. Role of *Pax6* in development of the cerebellar system. Development 126, 3585–3596 (1999). 10409504 10.1242/dev.126.16.3585

[b26] Osumi N., Shinohara H., Numayama-Tsuruta K. & Maekawa M. Concise review: Pax6 transcription factor contributes to both embryonic and adult neurogenesis as a multifunctional regulator. Stem Cells 26, 1663–1672 (2008). 18467663 10.1634/stemcells.2007-0884

[b27] Lin J. C. & Cepko C. L. Granule cell raphes and parasagittal domains of Purkinje cells: complementary patterns in the developing chick cerebellum. J. Neurosci. 18, 9342–9353 (1998). 9801373 10.1523/JNEUROSCI.18-22-09342.1998 PMC6792903

[b28] Dahmane N. & Ruiz i Altaba A. Sonic hedgehog regulates the growth and patterning of the cerebellum. Development 126, 3089–3100 (1999). 10375501 10.1242/dev.126.14.3089

[b29] Wallace V. A. Purkinje-cell-derived Sonic hedgehog regulates granule neuron precursor cell proliferation in the developing mouse cerebellum. Curr. Biol. 9, 445–448 (1999). 10226030 10.1016/s0960-9822(99)80195-x

[b30] Wechsler-Reya R. J. & Scott M. P. Control of neuronal precursor proliferation in the cerebellum by Sonic hedgehog. Neuron 22, 103–114 (1999). 10027293 10.1016/s0896-6273(00)80682-0

[b31] Xu H. *et al.* Bergmann glia function in granule cell migration during cerebellum development. Mol. Neurobiol. 47, 833–844 (2013). 23329344 10.1007/s12035-013-8405-y

[b32] Feng L., Hatten M. E. & Heintz N. Brain lipid-binding protein (BLBP): a novel signaling system in the developing mammalian CNS. Neuron 12, 895–908 (1994). 8161459 10.1016/0896-6273(94)90341-7

[b33] Soriano E., Alvarado-Mallart R. M., Dumesnil N., Del Río J. A. & Sotelo C. Cajal-Retzius cells regulate the radial glia phenotype in the adult and developing cerebellum and alter granule cell migration. Neuron 18, 563–577 (1997). 9136766 10.1016/s0896-6273(00)80298-6

[b34] Kuang Y. *et al.* Dicer and MiR-9 are required for proper Notch1 signaling and the Bergmann glial phenotype in the developing mouse cerebellum. Glia 60, 1734–1746 (2012). 22836445 10.1002/glia.22392

[b35] Brunne B. *et al.* Origin, maturation & astroglial transformation of secondary radial glial cells in the developing dentate gyrus. Glia 58, 1553–1569 (2010). 20549747 10.1002/glia.21029 PMC2946789

[b36] Stipursky J. & Gomes F. C. TGF-β1/SMAD signaling induces astrocyte fate commitment in vitro: implications for radial glia development. Glia 55, 1023–1033 (2007). 17549683 10.1002/glia.20522

[b37] Stipursky J., Francis D. & Gomes F. C. Activation of MAPK/PI3K/SMAD pathways by TGF-β(1) controls differentiation of radial glia into astrocytes in vitro. Dev. Neurosci. 34, 68–81 (2012). 22652705 10.1159/000338108

[b38] Schinstine M. & Lacovitti L. Expression of neuronal antigens by astrocytes derived from EGF-generated neuroprogenitor cells. Exp. Neurol. 141, 67–78 (1996). 8797669 10.1006/exnr.1996.0140

[b39] Shi Y. *et al.* Expression and function of orphan nuclear receptor TLX in adult neural stem cells. Nature 427, 78–83 (2004). 14702088 10.1038/nature02211

[b40] Frederiksen K. & McKay R. D. Proliferation and differentiation of rat neuroepithelial precursor cells in vivo. J. Neurosci. 8, 1144–1151 (1988). 3357014 10.1523/JNEUROSCI.08-04-01144.1988 PMC6569254

[b41] Dahlstrand J., Lardelli M. & Lendahl U. Nestin mRNA expression correlates with the central nervous system progenitor cell state in many, but not all, regions of developing central nervous system. Brain Res. Dev. Brain Res. 84, 109–129 (1995). 10.1016/0165-3806(94)00162-s 7720210

[b42] Anthony T. E., Mason H. A., Gridley T., Fishell G. & Heintz N. Brain lipid-binding protein is a direct target of Notch signaling in radial glial cells. Genes Dev. 19, 1028–1033 (2005). 15879553 10.1101/gad.1302105 PMC1091737

[b43] Gaiano N., Nye J. S. & Fishell G. Radial glial identity is promoted by *Notch1* signaling in the murine forebrain. Neuron 26, 395–404 (2000). 10839358 10.1016/s0896-6273(00)81172-1

[b44] Patten B. A., Peyrin J. M., Weinmaster G. & Corfas G. Sequential signaling through *Notch1* and erbB receptors mediates radial glia differentiation. J. Neurosci. 23, 6132–6140 (2003). 12853432 10.1523/JNEUROSCI.23-14-06132.2003 PMC6740346

[b45] Schmid R. S. *et al.* Neuregulin 1-erbB2 signaling is required for the establishment of radial glia and their transformation into astrocytes in cerebral cortex. Proc. Natl. Acad. Sci. USA 100, 4251–4256 (2003). 12649319 10.1073/pnas.0630496100 PMC153079

[b46] Gaiano N. & Fishell G. The role of notch in promoting glial and neural stem cell fates. Annu. Rev. Neurosci. 25, 471–490 (2002). 12052917 10.1146/annurev.neuro.25.030702.130823

[b47] Kopan R. & Ilagan M. X. G. The canonical Notch signaling pathway: unfolding the activation mechanism. Cell 137, 216–233 (2009). 19379690 10.1016/j.cell.2009.03.045 PMC2827930

[b48] Hu X., He W., Luo X., Tsubota K. E. & Yan R. BACE1 regulates hippocampal astrogenesis via the Jagged1-Notch pathway. Cell Rep. 4, 40–49 (2013). 23831026 10.1016/j.celrep.2013.06.005 PMC3740554

[b49] Park W. J. *et al.* Leucine-rich glioma inactivated 3 associates with syntaxin 1. Neurosci. Lett. 444, 240–244 (2008). 18760330 10.1016/j.neulet.2008.08.044

[b50] Leonardi E. *et al.* A computational model of the LGI1 protein suggests a common binding site for ADAM protein. PLoS One 6, e18142 (2011). 21479274 10.1371/journal.pone.0018142 PMC3066209

[b51] Reiss K. & Saftig P. The “a disintegrin and metalloprotease” (ADAM) family of sheddases: physiological and cellular functions. Semin. Cell Dev. Biol. 20, 126–137 (2009). 19049889 10.1016/j.semcdb.2008.11.002

[b52] Novak U. ADAM proteins in the brain. J. Clin. Neurosci. 11, 227–235 (2004). 14975408 10.1016/j.jocn.2003.10.006

[b53] Kunapuli P., Lo K., Hawthorn L. & Cowell J. K. Reexpression of *LGI1* in glioma cells results in dysregulation of genes implicated in the canonical axon guidance pathway. Genomics 95, 93–100 (2010). 19835947 10.1016/j.ygeno.2009.10.001 PMC2821952

[b54] Bray S. J. Notch signalling: a simple pathway becomes complex. Nat. Rev. Mol. Cell Biol. 7, 678–689 (2006). 16921404 10.1038/nrm2009

[b55] Wang D. J. *et al.* Long-term potentiation at cerebellar parallel fiber-Purkinje cell synapses requires presynaptic and postsynaptic signaling cascades. J. Neurosci. 34, 2355–2364 (2014). 24501374 10.1523/JNEUROSCI.4064-13.2014 PMC6608543

[b56] Li H., Berlin Y., Hart R. P. & Grumet M. Microtubules are critical for radial glial morphology: possible regulation by MAPs and MARKs. Glia 44, 37–46 (2003). 12951655 10.1002/glia.10267

[b57] Aoyama K., Matsumura N., Watanabe M. & Nakaki T. Oxidative stress on EAAC1 is involved in MPTP-induced glutathione depletion and motor dysfunction. Eur. J. Neurosci. 27, 20–30 (2008). 18093171 10.1111/j.1460-9568.2007.05979.x

[b58] Zhu J. *et al.* Chronic zinc exposure decreases the surface expression of NR2A-containing NMDA receptors in cultured hippocampal neurons. PLoS One 7, e46012 (2012). 23049922 10.1371/journal.pone.0046012 PMC3457937

[b59] Ji Y. F. *et al.* Upregulation of glutamate transporter GLT-1 by mTOR-Akt-NF-κB cascade in astrocytic oxygen-glucose deprivation. Glia 61, 1959–1975 (2013). 24108520 10.1002/glia.22566

[b60] Shao C. Y. *et al.* Distinct functions of nuclear distribution proteins LIS1, Ndel1 and NudCL in regulating axonal mitochondrial transport. Traffic 14, 785–797 (2013). 23551859 10.1111/tra.12070

