# Supplementary material for: Essential roles of leucine-rich glioma inactivated 1 in the development of embryonic and postnatal cerebellum
Source: Sci Rep. 2015 Jan 16;5:7827. doi: 10.1038/srep07827 (PMC4296302; doi:10.1038/srep07827)
Supplement: Supplementary Information — supplemental figures and legend [file srep07827-s1.pdf]

Essential roles of leucine-rich glioma inactivated 1 in the development of embryonic and postnatal cerebellum

Ya-Jun Xie, Liang Zhou, Nanwei Jiang, Nan Zhang, Na Zou, Lin Zhou,

Yin Wang, John K. Cowell, Ying Shen

Supplementary Information

Figure S1 | Phenotypic analysis of *Lgi1*-null mice. (**a**) Expression levels of LGI1 in the C57/B6 mouse cerebella during development. Equal amounts of protein from cerebellar extracts were analyzed by western blotting using antibodies against LGI1 and GAPDH. (**b**) Primers (F:5'-AGG ATG TGA TAC GTG GAA GA-3'; R:5'-CAC CCT GTT ACG TAT AGC CG-3') were used for the specific amplification of *Lgi1* in mouse genotyping. The expected 550 bp PCR band (left) was seen the cerebellum of WT mice, but not in KO mice. The predicted 64 KD LGI1 protein (right) was also recognized in WT mice using the anti-LGI1 antibody. (**c**) KO mice were smaller and had lower body weight. Averaged body weights were 4.9 ± 0.2 g (WT) and 4.1 ± 0.2 g (KO) at P7, n = 8 pairs, * $p < 0.05$ ($p = 0.023$, two-sided Student's *t*-test); and 8.6 ± 0.5 g (WT) and 6.6 ± 0.5 g (KO) at P14, n = 8 pairs, ** $p < 0.01$ ($p = 0.004$, two-sided Student's *t*-test). (**d**) The P14 KO mice exhibited a clasping behavior in the tail-suspension test. (**e**) The P14 KO cerebellum was slightly smaller than in the WT.

Figure S2 | Pax6 expression in the embryonic cerebellum. Total Pax6 expression was measured at E14.5, E15.5, E16.5, and E17.5, using GAPDH as the loading control. The

percentage changes of Pax6 (KO/WT) were 30.4 ± 7.5% (E14.5; $p = 0.00078$), 35.7 ± 6.9% (E15.5; $p = 0.00083$); 70.5 ± 10.2% (E16.5; $p = 0.034$), and 96.5 ± 10.3% (E17.5; $p = 0.34$). Data were derived from 3 pairs of mice. * $p < 0.05$. *** $p < 0.001$. n.s., no significance. One-sided Student's *t*-test.

Figure S3 | Expression levels of calbindin, Shh and Gli1 in the embryonic cerebellum. Total expression of Shh and Gli1 was decreased in embryonic KO cerebellum, but calbindin (calb) was not affected. GAPDH was the internal control. The percentage changes of Shh (KO/WT) were 23.1 ± 9.5% (E14.5; $p = 0.0056$), 28.8 ± 9.0% (E15.5; $p = 0.0076$), 35.6 ± 10.5% (E16.5; $p = 0.0087$), and 38.5 ± 13.5% (E17.5; $p = 0.0078$). The percentage changes of Gli1 (KO/WT) were 36.7 ± 9.8% (E14.5; $p = 0.0076$), 33.7 ± 11.3% (E15.5; $p = 0.0086$), 38.3 ± 10.9% (E16.5; $p = 0.0094$), and 37.9 ± 12.4% (E17.5; $p = 0.0089$). The percentage changes of calbindin (KO/WT) were 90.5 ± 6.7% (E14.5; $p = 0.36$), 96.8 ± 8.7% (E15.5; $p = 0.34$), 99.2 ± 7.4% (E16.5; $p = 0.42$), and 96.4 ± 9.8% (E17.5; $p = 0.38$). Data were derived from 3 pairs of mice. ** $p < 0.01$. n.s., no significance. One-sided Student's *t*-test.

Figure S4 | Expression levels of BLBP and NICD in the embryonic cerebellum. GAPDH was the internal control. The percentage changes of BLBP (KO/WT) were 99.5 ± 6.7% (E14.5; $p = 0.40$), 102.4 ± 7.3% (E15.5; $p = 0.11$), 108.3 ± 7.6% (E16.5; $p = 0.083$), and 118.4 ± 6.9 % (E17.5; $p = 0.031$). The percentage changes of NICD (KO/WT) were 92.4 ± 6.3% (E14.5; $p = 0.37$), 94.3 ± 7.8% (E15.5; $p = 0.43$), 102.5 ± 5.4% (E16.5; p =

0.39), and 101.2 ± 6.7% (E17.5; $p = 0.47$). Data were derived from 3 pairs of mice. * $p < 0.05$. n.s., no significance. One-sided Student's *t*-test.

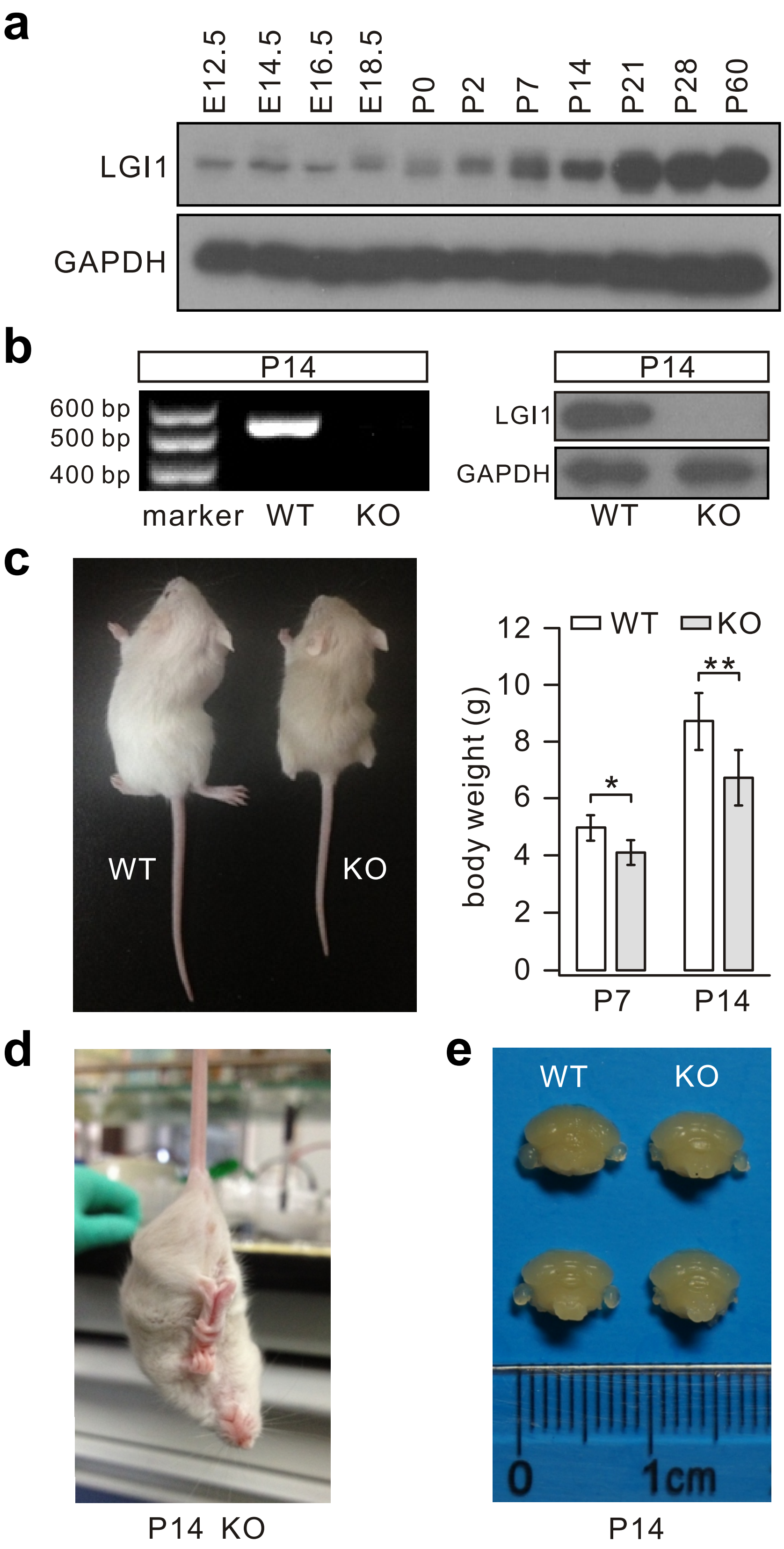
a
E12.5
E14.5
E16.5
E18.5
P0
P2
P7
P14
P21
P28
P60
LGI1
GAPDH
b
P14
600 bp
500 bp
400 bp
marker
WT
KO
P14
LGI1
GAPDH
WT
KO
c
WT
KO
body weight (g)
12
10
8
6
4
2
0
WT
KO
*
**
P7
P14
d
P14 KO
e
WT
KO
0
1cm
P14

Figure S1

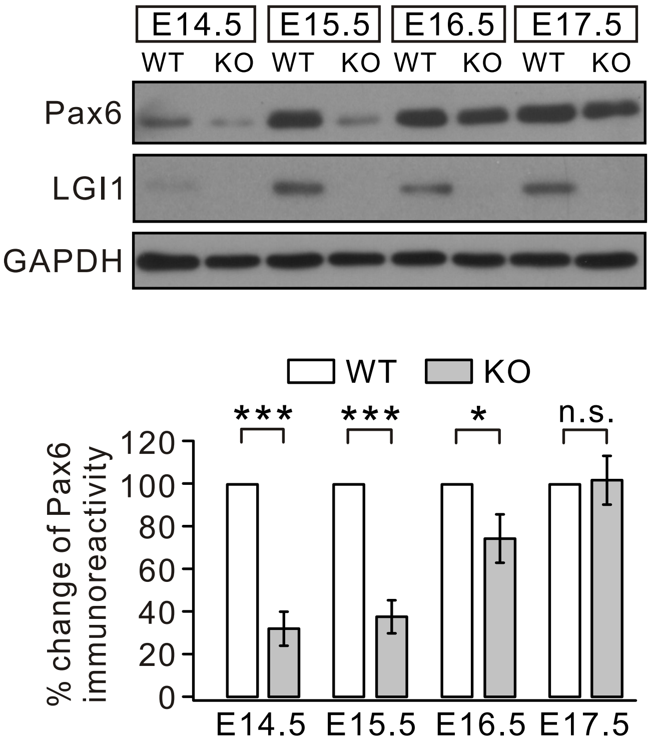
E14.5
E15.5
E16.5
E17.5
WT
KO
Pax6
LGI1
GAPDH
WT
KO
***
***
*
n.s.
% change of Pax6 immunoreactivity
0
20
40
60
80
100
120
E14.5
E15.5
E16.5
E17.5

Figure S2

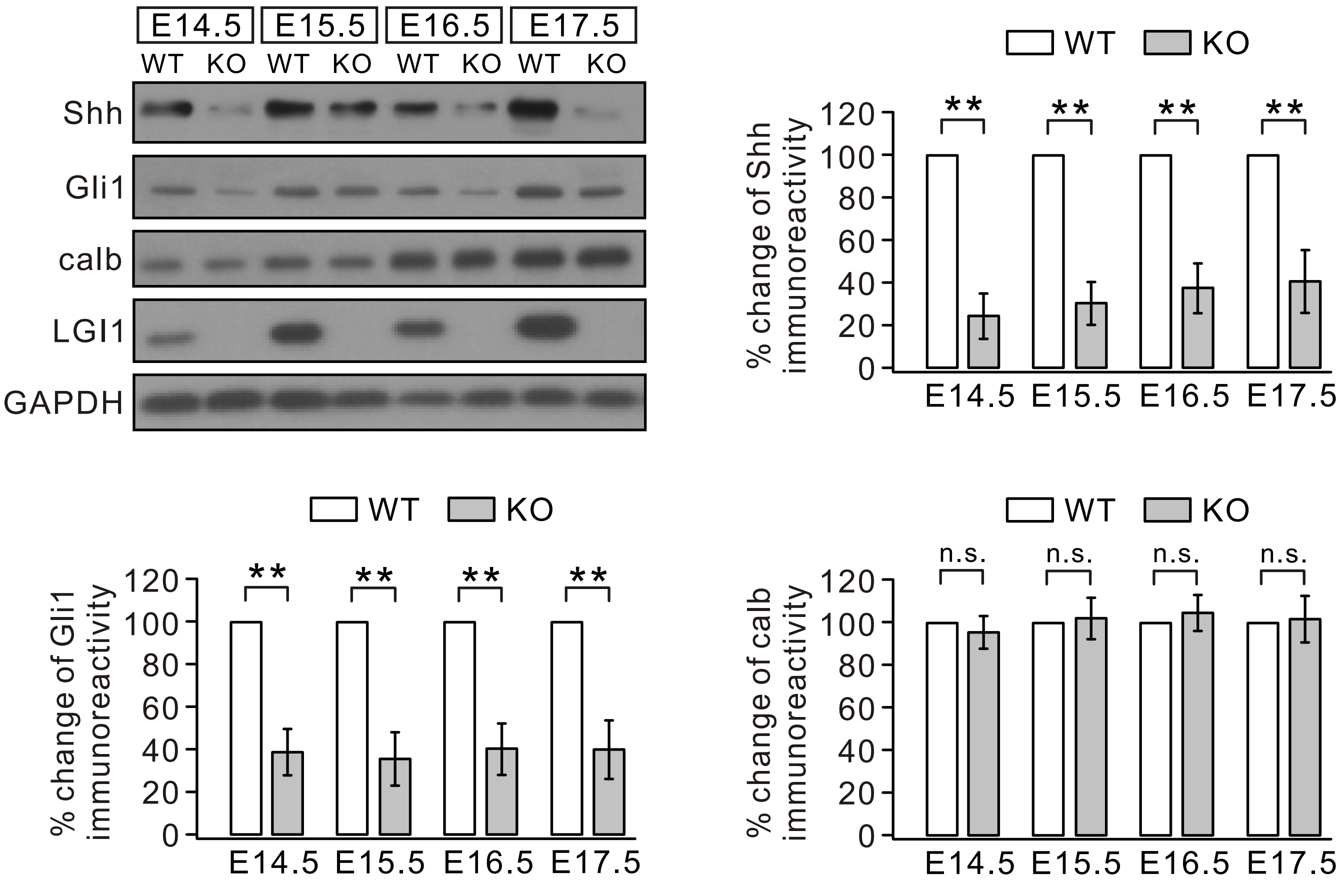

E14.5
E15.5
E16.5
E17.5
WT KO WT KO WT KO WT KO
Shh
Gli1
calb
LGI1
GAPDH
WT KO
% change of Shh immunoreactivity
% change of Gli1 immunoreactivity
% change of calb immunoreactivity
**
n.s.
0 20 40 60 80 100 120

Figure S3

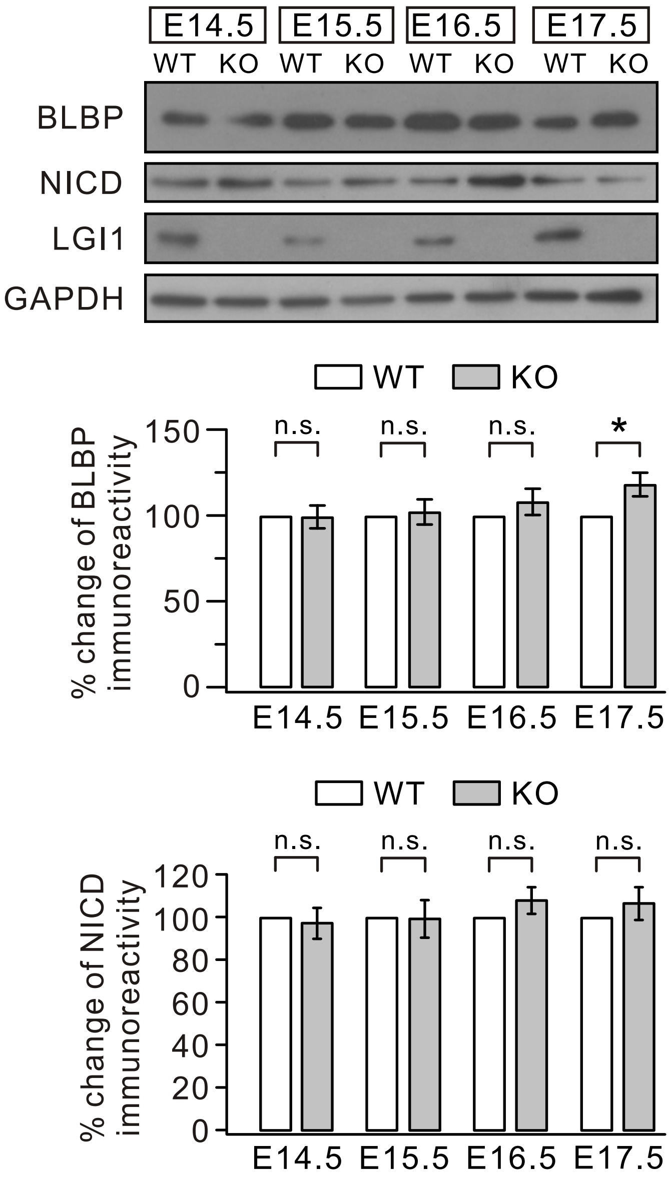
E14.5
E15.5
E16.5
E17.5
WT KO WT KO WT KO WT KO
BLBP
NICD
LGI1
GAPDH
WT
KO
% change of BLBP immunoreactivity
150
100
50
0
n.s.
n.s.
n.s.
*
E14.5 E15.5 E16.5 E17.5
WT
KO
% change of NICD immunoreactivity
120
100
80
60
40
20
0
n.s.
n.s.
n.s.
n.s.
E14.5 E15.5 E16.5 E17.5

Figure S4
